# Supplementary material for: Patterns of Intron Gain and Loss in Fungi
Source: PLoS Biol. 2004 Nov 30;2(12):e422. doi: 10.1371/journal.pbio.0020422 (PMC532390; doi:10.1371/journal.pbio.0020422)
Supplement: Table S1 — Also available at http://genes.mit.edu/NielsenEtAl/. (4.3 MB ZIP). [file pbio.0020422.st001.zip › NielsenEtAl/html/1197.html]

AN4469.1.NCU03764.1.MG02460.1.FG06821.1


```
 CLUSTAL W (1.82) Multiple Sequence Alignments - Introns Inserted


Sequence 1: MG02460.1	531 aa
Sequence 2: FG06821.1	475 aa
Sequence 3: NCU03764.1	487 aa
Sequence 4: AN4469.1	485 aa
Alignment Length: 548 aa
Number Identitical Residues: 168 aa
Alignment Score (without introns) 9435


MG02460.1 	MN------ATTEQEAIVKVLFTTTEQGLELPESKRLLLVPAD1IRRYGLSRVLNSESMLN
NCU03764.1	MD------HPTESGAQVRVTFTTQEEDIQLDESKRQLLVPAD~IRRYGLSRILNSESMLD
FG06821.1 	MG------DATQ----VKVVFTTTEQDLVLPESKQQLLVPAD1IKRYGLSRILNSESMLD
AN4469.1  	MDTSQVSVSSQAAQRQVRVQLTSKQEDIALPDNTGPILVPTG1LKRYALSTLVNN--LLG
          	*.::. :  .      *:* :*: ::.: * :..  :***:. ::**.** ::*.  :*.

MG02460.1 	TAAPIPFDFLVNGTFLRTTLEDYLKENGLPFEKTVTLQYVRSLVPPAYEASFEHDDWVSS
NCU03764.1	TNS-IPFDFLVNGSFLRGSLEEYLNANGLSLETNITLQYVRSLIPPTYEASFEHDDWVSA
FG06821.1 	TSSPVPLDFLANGTFLRTSIEEYLATHGLSSESTLTLQYVRSLLPPVYEASFEHDDWVSG
AN4469.1  	NDKPIPFEFLINGSFLRTSIDEYLTANGISAETTLEIEYVRALIPPLHIASFEHDDWVSS
          	.  .:*::** **:*** ::::**  :*:. *..: ::***:*:** : **********.

MG02460.1 	VDLLSATSSAGKWSGSSFLQGQDRILSASYDGLLRIWNGSGQALATSSVVNRGPLCG-LK
NCU03764.1	VDVLSATSPAGRWSGDNFQRGQDRILSASYDGLLRIWDASGNVIGTSPSGSHGGHTASIK
FG06821.1 	VDVLSATSRAGLLAGGANVP--ERVASSSYDGLVRIWNPSGDAIAVSPAGRAGGHTQRAN
AN4469.1  	IDVLSTSSPASAGS-DAIARGQERILSGSYDGFLRVWNMSSQVIATSPSPTDGGHISSIK
          	:*:**::* *.  : .    ..:*: *.****::*:*: *.:.:..*.    *      :

MG02460.1 	SAKFMSSTKIAAAGLDRTVRIWDYTEADDHFS--GQLKPTLELYGHRSIIESLGVDGSSR
NCU03764.1	AAKFLSSTQIASAGMDRTVRVWKYTESGESYERRGELKPTLELYGHTSSIDSLEVDGASK
FG06821.1 	VVKWISQKQLASAGLDRKVIVWDYSESDDGFS--GSLKPSMELWGHEKDINSIDVNGATK
AN4469.1  	AAKFISPSSIASAGLDRTVRLWKYTEAEDGFS--GKIVPQVELYGHKSGINSLAVHASTN
          	 .*::* ..:*:**:**.* :*.*:*: : :.  *.: * :**:** . *:*: *..::.

MG02460.1 	RILTACADGSIGLWTTSKKLAPEAP-TALLPQTYATKRRKGSTTSANIIAQRGALSLIPV
NCU03764.1	RILTASADGSIGFWTTSKASAPEVSDSSLLPGAHTSKRRK--LATSVTAAQRGPLALMNI
FG06821.1 	RILTASSDGKVGLWTSSKRTAPQAD-SESLPSAHSTKRAK--LASAANTAQRGPLALISV
AN4469.1  	RILSASADHNVGFWSTKKSDAPAAP-ESLLPSAASRTSKRRKLNASVSVSQRGPLALLSG
          	***:*.:* .:*:*::.*  ** .     ** : : .  : .  ::   :***.*:*:  

MG02460.1 	HSRPVSAAIFDPQNQHTAYSASQDHTLKVLDLTTSRVVSTITTSNALMSACYLSNK--SA
NCU03764.1	HNAPATAAVFDPRDRTVAYSVSQDHTVKTIDLTTSSVVSTFTTSHALLSLAALPRSSASA
FG06821.1 	HDEPVTAAIFHPNDATVAYSASKDHTVRTIDLTTQREVSRLTTMHPLLCATALP----GS
AN4469.1  	HTAPVSDAIFDARDSTVGYSVSWDHSLRTWDLVTAALVDTRTTSHSLLSLQHLP----DH
          	*  *.: *:*...:  ..**.* **:::. **.*   *.  ** :.*:.   *.    . 

MG02460.1 	PLIAAGTSSRNVTLIDPRESAAATSVMTLRGHINVVSSVSASPDNEYSLVSGSHDGTCRI
NCU03764.1	PLLACGTSARHITLVDPRTSTAATSVMTLRGHANKVVSLAANPENEYSLVSGSHDGTCRI
FG06821.1 	SLIAAGSSARHITLLDPRESASSTSAMTLRGHVNMVVSLSASPENDYSLVSGSHDSTCRV
AN4469.1  	NLLATGTSARHITLIDPRASAATISAMTLRGHTNAVVSLARDPHSIYGLISGSHDGTCRI
          	 *:* *:*:*::**:*** *::: *.****** * * *:: .*.. *.*:*****.***:

MG02460.1 	WDLRSVRPAAGKEETGLGSVGESVYLIERESLG--DKKR--PLAGEGVKVFDVQWDKTWG
NCU03764.1	WDLRSVRP-ATKDEGGMGSVSEPVYVIDRESQQ--GKKKKSSVTGDGCKVFSVVWDQ-LG
FG06821.1 	WDLRSTR--MGNSEEGGGSVSEPVYTIGREWLK--GKKL--PPAGDGAKVLGVSWDKTWG
AN4469.1  	WDLRATK-----TDKG-GAVGESVYSISRKSLEEEGKANSKRVGGEGVKVFSVCWDREVG
          	****:.:      : * *:*.*.** * *:   ...*  ..   *:* **:.* **:  *

MG02460.1 	I0PSK-EVPQLPSRKYHVYYETGNLIVSNFNYHQVQLLNRLLCIVHNRREPKAWIKHFKP
NCU03764.1	I~FSGGEDKKVQINKGRGVNAE--------------------------------------
FG06821.1 	I~VSGGEDKKVQINRGRDLFGSGS------------------------------------
AN4469.1  	I~VSAGEDKRIQINRGEGVLSSS-------------------------------------
          	*  * .*  ::  .: .     .                                     

MG02460.1 	RRLPPTVVVK
NCU03764.1	----------
FG06821.1 	----------
AN4469.1  	----------
          	
```
